# National Dental Association

**Published:** 1899-01

**Authors:** 


### Reports of Society Meetings.

### NATIONAL DENTAL ASSOCIATION.

(Continued from Vol. XIX., page 817.)

### THE COMPARATIVE METHOD OF TEACHING DENTAL ANATOMY.[1]

THE wonderful collection of skulls and teeth exhibited at the Old Point meeting of this Association and the papers read bearing upon the subject afford gratifying proof of the growing interest in the subject of odontography, a branch of study which has great value as a contributory science, and which should be recognized in

[1] Abstract of paper read by Dr. A. H. Thompson.

the college curriculum as throwing light upon human odontology. The limited human denture has long been studied as comprising the whole range of the subject as taught in our schools, all the organs of the human body being studied as though man were a special creation, independent of all other forms of life. But this has all been changed; all life is now regarded as a unit, and man as but a very insignificant part of the realms of nature. He has no *kingdom* of his own; he is a vertebrate, like other vertebrates. He has no *class* of his own; he is a mammal, like other mammals. He has no *order* of his own; he is a primate, sharing this distinction with apes and monkeys; in fact, the skeleton of the higher apes resembles that of man more closely than that of the monkeys. The organizations of the lower animals should be studied in connection with that of man, for the knowledge to be gained by comparison of related types of structure. The paths of evolution have been marked out, and the life-history of the lower animals is now well understood. The story of the evolution of the type of man from the lowest forms has been revealed only through study by the comparative method. The human embryo recalls types of the lower forms at various stages of its development. This is also true of the various organs, of which much is learned through the study of their evolution, from the lowest organisms in which a suggestion of these organs may be found to the highest types.

The comparative method having been applied to the study of other organs, it is but rational that it should be similarly employed in the study of the teeth of man. The life-history of the teeth is of special interest to us as dentists, and we should consider it from the scientific stand-point.

The teeth are developed for a functional purpose,—that is, the reduction of food preparatory to digestion. From the study of the teeth of lower animals we learn that, morphologically, they are mere dermal structures, modified and elaborated for food-reducing purposes.

The variety of jaw movements is important from the influence that this force has upon the form and size of the jaws and masticatory apparatus, and its effect upon the forms and positions of the teeth. The adaptation of tooth-forms to the various kinds of food is wonderful and beautiful, and the various types illustrate the variations in the forms of the teeth as adapted to their varied functions: the incisors for cutting purposes; the canines as prehensile organs of extraordinary development and form in some of the lower animals, but greatly reduced in man; the molars as crushing and masticating organs of greatly varying forms, adapted to various kinds of food.

The evolution of the jaws and of jaw-movements, and the principles of occlusion, a thorough knowledge of which is of such vital importance to us in our every-day operations, are greatly in need of illumination. We have studied only the jaws of man, which are but rudimentary as compared with the jaws of many other animals. We need to learn more of the mechanism of the jaws from animals in whom it is more highly specialized.

The relation of tooth-forms to jaw-movement is a most interesting branch of the subject, there being an exact relationship between tooth-forms and their functions and jaw-movements. It is a field which offers to the student great possibilities of discovery.

DISCUSSION.

In the discussion of this paper Dr. Barrett said that mastication of food is *not* the first function of the teeth; they are primarily weapons of offence and defence; secondarily, they are organs of prehension; thirdly, *perhaps*, they are organs of mastication.

Dr. Crawford cannot agree with Dr. Barrett that the human teeth were primarily organs of offence and defence. These questions should be studied from the stand-point of nature, in their bearings upon life, character, and civilization.

Dr. Thompson, in closing the discussion, said that he believes food-reduction for the nutrition of the system to be the primary function of the teeth, their function as weapons or tools being secondary, not primary. He referred to Dr. Walker's studies of the teeth and jaw-movements as having thrown much light upon the subject; he hoped he would do more work in this direction.

There being no lantern available for the exhibition of Dr. Cryer's slides, his paper was not read.

Section VIII. was not represented.

Section IX. offered one paper, by Dr. C. S. Case, entitled "Answer to the Criticism of my Paper entitled 'Principles of Force and Anchorage in the Movement of Teeth,'" of which we here present a brief abstract, as complete as possible, without the illustrations, models, and apparatus which accompanied the paper.

Dr. Case referred to the controversy of individual opinion which had arisen last year at the meeting of the American Dental Association, as to the action of force when applied to an extension above the gingival margin of the crown of a rigid bar made integral with the crown of the tooth.

Dr. Case's methods are based upon the principle that when a force is applied anywhere upon such an extension, above and on the outside of the gum, it will have exactly the same effect as if applied to the root at a point on a line with the direction of said force.

To place beyond controversy this claim (which was disputed last year), Dr. Case had constructed an apparatus consisting of a large wooden tooth, suspended in an upright position by being attached along the posterior surface of the tooth to five steel spiral springs, which in turn are fastened to and supported by a frame.

These springs were so adjusted that a given power applied to each separately would produce the same amount of motion in compression or extension.

Force applied to this tooth in a manner similar to that which is possible with a dental regulating apparatus represents the direction and, approximately, the energy expended at different locations on the root, the movement being registered by hands on dials. To the anterior surface of the crown was an attachment, with an extension above the cervical portion, similar to that used by Dr. Case, with notches at different points for the attachment of power bars.

Power applied at the highest point upon the crown possible with an ordinary regulating appliance tipped the tooth from an upright position, the cervical portion moving in the direction of the applied power, the apical portion moving about one-fifth as far in the opposite direction.

Power applied at various points above the cervical margin found a place where the end of the root ceased to move in the opposite direction, remaining stationary as a pivotal point to movement in other parts.

Power applied upon the upright bar, at a point whose line of force intersects the root in the centre of its area of resistance, moved the whole tooth in the direction of the force.

The apparatus thus proves that force acting upon a bar made rigid with the crown and extending in front of the gum will produce the same effect as if applied to the root itself, and the same as if the space between the bar and the root were one solid mass, which was proved in the apparatus by inserting a properly shaped piece of wood, filling the space. The principle contended for was demonstrated by the apparatus, though allowance must, of course, it for the reception of a tap-bolt. The collar must be well contoured to fit the surface of the tooth. Several methods of drawing the collar tight around the tooth have been utilized. For most cases the tap-bolt and nut give the most satisfaction. They are made of hard, non-corrosive metal, and provided with a deep, strong thread, with the thickness of the nut about one-half or more of the lateral diameter of the tooth. In some cases funnel-shaped tubes are used for the reception of conical nuts. The bolt may enter from either the buccal or lingual side. A strong clip of plate-metal is made to extend from the collar to the grinding surface of the abutment tooth, to prevent the collar from slipping towards the gum. The clip should be stiff enough to withstand any pressure that may be made in mastication.

If the bridge is to rest in contact with the gum, a saddle of thin platinum, or platinum and gold, is swaged to fit the gum. When the collars and saddle have been accurately fitted to the model, porcelain teeth are backed, articulated, and waxed into place, and the parts united as in ordinary bridge-work; or, the collars and saddle having been adjusted, plain rubber teeth may be used and the case packed with rubber and vulcanized as in making a partial rubber plate. In suitable cases the Mason detachable porcelain crowns may be used.

For crossing the labial side of a cuspid, a loop of gold wire, flattened on the under side, may be used, marring the appearance less than a bar or narrow collar; the mesial, distal, and lingual surfaces of the cuspid should be well covered with a cap. One end of the gold wire should be threaded to pass through a tube and engage with the nut; the other end may be bent at a right angle to form a hook to catch in a hole on the distal side.

Before setting the bridge the teeth for anchorage are polished and made dry, and the inner side of the collars covered with a thick solution of chloro-percha. The collars are sprung backward as the bridge is adjusted; they are then drawn around the teeth and the tap-bolts screwed quickly to place. After the bridge has been made firm, the small openings around the heads of the screws may be filled with gutta-percha. In some cases a bolt with roughened surface can be retained with oxyphosphate cement, not depending alone on the screw-thread.

Dr. Jackson described in detail a large number of cases, and exhibited a number of models, with these bridges in position, showing the various applications of this method.

The paper was passed without discussion.

RESOLUTIONS AND OTHER MISCELLANEOUS ITEMS.

In regard to the proposed amendment to the patent law:

WHEREAS, The Supreme Court having already declared all such patents, as are contemplated in this move to amend the patent law, invalid, which makes all such amendments unnecessary; and

WHEREAS, The constant agitation of this question is detrimental to the best interests of organization in existence, be it

*Resolved*, That the National Dental Association, now in session, disapproves of any further work in this direction, and recommends that the whole question be dropped as unwise and unnecessary.

The following resolutions were offered by Dr. C. L. Goddard, in relation to the appointment of dental surgeons to the army and navy:

*Resolved*, That this Association approves and endorses the movement for the appointment of dentists in the army.

*Resolved*, That a committee of five be appointed by the president to represent this Association in procuring from Congress the necessary legislation.

The resolutions were referred to the Executive Committee, which, at a later session, reported as follows:

WHEREAS, The National Dental Association has appointed a committee to take charge of and have full control of the subject of legislation concerning the employment of dental surgeons in the army and navy,

*Resolved*, That this Association deprecates any independent action on the part of State and local societies or individuals without the approval of said committee.

Adopted.

The committee appointed consists of Drs. Finley, Donnally, and Butler.

In regard to the International Dental Congress, Paris, 1900, the following resolution was offered by Dr. A. W. Harlan and unanimously adopted:

WHEREAS, Provision has been made for holding an international dental congress in Paris, and committees have already been appointed to organize such a congress, and that it is the will of said committees that the National Dental Association appoint a committee to co-operate with those committees in making the congress a success, therefore be it

*Resolved*, That a general committee of fifteen be appointed by the president to co-operate with the General Committee in Paris, such committee to have power to add to its number, not to exceed twenty-five names in the United it for the reception of a tap-bolt. The collar must be well contoured to fit the surface of the tooth. Several methods of drawing the collar tight around the tooth have been utilized. For most cases the tap-bolt and nut give the most satisfaction. They are made of hard, non-corrosive metal, and provided with a deep, strong thread, with the thickness of the nut about one-half or more of the lateral diameter of the tooth. In some cases funnel-shaped tubes are used for the reception of conical nuts. The bolt may enter from either the buccal or lingual side. A strong clip of plate-metal is made to extend from the collar to the grinding surface of the abutment tooth, to prevent the collar from slipping towards the gum. The clip should be stiff enough to withstand any pressure that may be made in mastication.

If the bridge is to rest in contact with the gum, a saddle of thin platinum, or platinum and gold, is swaged to fit the gum. When the collars and saddle have been accurately fitted to the model, porcelain teeth are backed, articulated, and waxed into place, and the parts united as in ordinary bridge-work; or, the collars and saddle having been adjusted, plain rubber teeth may be used and the case packed with rubber and vulcanized as in making a partial rubber plate. In suitable cases the Mason detachable porcelain crowns may be used.

For crossing the labial side of a cuspid, a loop of gold wire, flattened on the under side, may be used, marring the appearance less than a bar or narrow collar; the mesial, distal, and lingual surfaces of the cuspid should be well covered with a cap. One end of the gold wire should be threaded to pass through a tube and engage with the nut; the other end may be bent at a right angle to form a hook to catch in a hole on the distal side.

Before setting the bridge the teeth for anchorage are polished and made dry, and the inner side of the collars covered with a thick solution of chloro-percha. The collars are sprung backward as the bridge is adjusted; they are then drawn around the teeth and the tap-bolts screwed quickly to place. After the bridge has been made firm, the small openings around the heads of the screws may be filled with gutta-percha. In some cases a bolt with roughened surface can be retained with oxyphosphate cement, not depending alone on the screw-thread.

Dr. Jackson described in detail a large number of cases, and exhibited a number of models, with these bridges in position, showing the various applications of this method.

The paper was passed without discussion.

RESOLUTIONS AND OTHER MISCELLANEOUS ITEMS.

In regard to the proposed amendment to the patent law:

WHEREAS, The Supreme Court having already declared all such patents, as are contemplated in this move to amend the patent law, invalid, which makes all such amendments unnecessary; and

WHEREAS, The constant agitation of this question is detrimental to the best interests of organization in existence, be it

*Resolved*, That the National Dental Association, now in session, disapproves of any further work in this direction, and recommends that the whole question be dropped as unwise and unnecessary.

The following resolutions were offered by Dr. C. L. Goddard, in relation to the appointment of dental surgeons to the army and navy:

*Resolved*, That this Association approves and endorses the movement for the appointment of dentists in the army.

*Resolved*, That a committee of five be appointed by the president to represent this Association in procuring from Congress the necessary legislation.

The resolutions were referred to the Executive Committee, which, at a later session, reported as follows:

WHEREAS, The National Dental Association has appointed a committee to take charge of and have full control of the subject of legislation concerning the employment of dental surgeons in the army and navy,

*Resolved*, That this Association deprecates any independent action on the part of State and local societies or individuals without the approval of said committee.

Adopted.

The committee appointed consists of Drs. Finley, Donnally, and Butler.

In regard to the International Dental Congress, Paris, 1900, the following resolution was offered by Dr. A. W. Harlan and unanimously adopted:

WHEREAS, Provision has been made for holding an international dental congress in Paris, and committees have already been appointed to organize such a congress, and that it is the will of said committees that the National Dental Association appoint a committee to co-operate with those committees in making the congress a success, therefore be it

*Resolved*, That a general committee of fifteen be appointed by the president to co-operate with the General Committee in Paris, such committee to have power to add to its number, not to exceed twenty-five names in the United

States; and that said committee, when organized, be empowered to adopt rules and regulations such as will insure the success of the congress. The present president and vice-presidents of this body shall be members of this committee. After this committee has organized by the selection of a chairman and secretary, its acts shall be final, and, after the close of the congress, said committee shall present a report of its work to this Association.

At a subsequent session, the committee of fifteen, together with the president and three vice-presidents, was appointed, as follows: A. W. Harlan, Chicago; A. H. Fuller, St. Louis; H. J. McKellops, St. Louis; J. Taft, Cincinnati; H. A. Smith, Cincinnati; W. W. Walker, New York; James McManus, Hartford; W. C. Barrett, Buffalo; T. W. Brophy, Chicago; B. Holly Smith, Baltimore; W. E. Griswold, Denver; C. L. Goddard, San Francisco; L. L. Dunbar, San Francisco; H. W. Morgan, Nashville; Frank Holland, Atlanta; E. C. Kirk, Philadelphia; J. D. Patterson, Kansas City, Mo.; Thomas Fillebrown, Boston; Thomas E. Weeks, Minneapolis.

The Committee on the President's Address having recommended the adoption of a code of ethics, the chair appointed Drs. H. A. Smith (who was associated with Dr. George Watts in the formulation of the code of ethics by which the American Dental Association was governed for so many years), Frank Holland, and H. W. Morgan a committee to formulate a code of ethics.

Chairmen and secretaries of sections:

Section I.—I. N. Broomell, chairman, William E. Walker, secretary.

Section II.—S. H. Guilford, chairman; M. F. Finley, secretary.

Section III.—J. Y. Crawford, chairman; Frank Holland, secretary.

Section IV.—T. L. James, chairman; L. L. Dunbar, secretary.

Section V.—J. S. Cassidy, chairman: A. W. Harlan, secretary.

Section VI.—J. D. Patterson, chairman; L. E. Custer, secretary.

Section VII.—W. C. Barrett, chairman; W. F. Lewis, secretary.

Section VIII.—J. Taft, chairman; H. R. McFadden, secretary.

Section IX.—V. H. Jackson, chairman; C. L. Goddard, secretary.

Section X.—H. J. McKellops, chairman; M. B. Culver, secretary.

Dr. A. W. Harlan offered the following *standing resolutions*, which were unanimously adopted:

*Resolved*, That the secretaries of sections are required to forward to the chairman of the Executive Committee, sixty days before the annual meeting, the titles of papers to be submitted, with the names of the authors, in order that they may appear on the official programme.

*Resolved,* That the Executive Committee shall prepare and mail to the members an official programme at least twenty days before the annual meeting. This programme shall have a list of the hotels, the place of meeting, and the railway and steamboat facilities for getting to the place of meeting from the principal points in the United States. This notice shall be mailed to the journals at least one month before the annual meeting.

Dr. Molyneaux offered the following:

*Resolved,* That a committee of three be appointed, who shall prepare amendments to our constitution providing for an executive council, to whom all miscellaneous business shall be submitted without discussion before final action is taken.

Adopted.

Dr. Cassidy offered as a standing resolution, which was adopted, the following:

*Resolved,* That the outgoing president be a member of the Executive Council.

The chair appointed as the other two members of the Executive Council for the coming year Drs. Grant Molyneaux and John S. Marshall.

Result of the elections:

President (from the East).—Dr. H. J. Burkhart, Batavia. N. Y.

Vice-president (from the East).—Dr. S. H. Guilford, Philadelphia.

Vice-president (from the West).—Dr. Thomas E. Weeks, Minneapolis.

Vice-president (from the South).—Dr. B. Holly Smith, Baltimore.

(Age alone confers seniority among the vice-presidents.)

The following officers were all unanimously re-elected to their several offices:

George H. Cushing, recording secretary.

William Ernest Walker, assistant recording secretary.

Emma Eames Chase, corresponding secretary.

Drs. G. V. I. Brown, C. S. Butler, and J. Y. Crawford were elected members of the Executive Committee in the places of Drs. C. N. Peirce, W. P. Dickinson, and George Eubank, whose terms expire with the present meeting.

Niagara Falls and Boston were placed in nomination for the place of next meeting, Niagara Falls being chosen. By a unanimous vote the time of meeting was changed to the first Tuesday in August.

The Executive Committee appointed Dr. L. E. Custer, of Dayton, Ohio, to deliver a general address on "Electricity," and Drs. T. W. Brophy and Thomas Fillebrown to deliver an address on "The Surgical Treatment of Cleft Palate."

Publication Committee: C. N. Johnson, Chicago; C. N. Peirce, Philadelphia.

Local Committee of Arrangements: C. A. Butler, D. F. Bentley, Buffalo, and M. O. Cooley.

Committee on National Museum: Williams Donnally, Washington City, Henry W. Morgan, Nashville, and John S. Marshall, Chicago.

AMENDMENTS TO THE CONSTITUTION.

The proposed amendments to the constitution, laid over from last year, (1) by Dr. J. Y. Crawford, to change the name of the Association to "The American Association of Dental Surgeons;" (2) by Dr. S. H. Guilford, to change the name to "The American Association of Dental Science;" and (3) by Dr. Stellwagen, to "The American Stomatological Association," were all tabled.

The following amendments, proposed by Dr. M. F. Finley, lay over for one session, and were then unanimously adopted:

Amend Section 1, Article IV., by inserting after the third word, first line, "and the societies bearing the names of the Territories enumerated in Section 3, Article XII."

Amend Section 3, Article III., by inserting after the first word of the third line, "and Territorial."

The following amendment, offered by Dr. H. J. Burkhart, lay over one session, and was then unanimously adopted:

Amend Article VI., second paragraph of Section 3, to read, "commencing with 1897, the president shall be chosen from the division in which the next meeting is to be held."

The following amendments, embodying the suggestions in the president's address and recommended by the Committee on the Address, were read by Dr. H. W. Morgan, but were not acted upon:

Art. VI. shall be amended so as to read, "After 1898 the president shall be chosen from the division in which the next annual meeting is to be held."

Article III., Section 3, shall be changed so as to read as follows: "They shall be chosen in any manner that their Association may see proper."

In Article IV., Section 1, the word ten shall be changed so as to read "six."

The following amendments, offered by Dr. Laurence Leonard, lie over for one year:

"*Resolved*, That Article V. be amended by adding after the word dollars, "the receipt for which will entitle the holder to all the privileges of the floor, including general meetings of the Executive Committee."

Article VIII., Section 1, after the words "Association to whom," add "and before whom all business matters shall come."

Article VIII., create Section 10, "The Executive Committee shall elect a president and secretary, and shall hold daily general sessions during the meeting of the Association."

Create Article XV. as follows: Section 1. After 1899 nothing shall come before the general sessions of the Association except the president's address, announcements, election of officers, selection of place of meeting, and the reading and discussion of scientific papers endorsed by the sections.

Section 2. Anything in conflict with this article is hereby repealed.

A communication was received from the Northern Illinois Dental Society, reporting the adoption by that body of resolutions looking towards a remedy for the existing evils regarding the interstate practice of dentistry, and requesting the National Dental Association to work in securing such modifications of the dental laws of the various States as shall enable competent practitioners to remove from one State to another without being compelled to submit to provisions which are eminently unfair to large numbers of capable dentists.

The communication was referred to Section 2.

Adjourned to meet at Niagara Falls the first Tuesday in August, 1899.

